# Recent Advances in Wide-Range Temperature Metal-CO_2_ Batteries: A Mini Review

**DOI:** 10.1007/s40820-024-01607-x

**Published:** 2024-12-30

**Authors:** Xuejing Zhang, Ning Zhao, Hanqi Zhang, Yiming Fan, Feng Jin, Chunsheng Li, Yan Sun, Jiaqi Wang, Ming Chen, Xiaofei Hu

**Affiliations:** 1 School of Environmental and Chemical Engineering, Jiangsu University of Science and Technology, Zhenjiang, 212003 People’s Republic of China; 2 Engineering Research Center of Energy Storage Materials and Devices, Ministry of Education, School of Chemistry, Xi’an Jiaotong University, Xi’an, 710049 People’s Republic of China; 3 State Key Laboratory for Strength and Vibration of Mechanical Structures, Xi’an Jiaotong University, Xi’an, 710049 People’s Republic of China; 4 Key Laboratory of Advanced Electrode Materials for Novel Solar Cells for Petroleum and Chemical Industry of China, School of Chemistry and Life Sciences, Suzhou University of Science and Technology, Suzhou, 215009 People’s Republic of China; 5 Department of Chemistry and Biochemistry, Northern Illinois University, DeKalb, IL 60115 USA; 6 School of Chemistry and Chemical Engineering, Yangzhou University, Yangzhou, 225002 People’s Republic of China

**Keywords:** M-CO_2_ batteries, Wide-range temperature, Electrolytes, Interfaces, Electrode reactions

## Abstract

The metal–carbon dioxide batteries, emerging as high-energy–density energy storage devices, enable direct CO_2_ utilization, offering promising prospects for CO_2_ capture and utilization, energy conversion, and storage. However, the electrochemical performance of M-CO_2_ batteries faces significant challenges, particularly at extreme temperatures. Issues such as high overpotential, poor charge reversibility, and cycling capacity decay arise from complex reaction interfaces, sluggish oxidation kinetics, inefficient catalysts, dendrite growth, and unstable electrolytes. Despite significant advancements at room temperature, limited research has focused on the performance of M-CO_2_ batteries across a wide-temperature range. This review examines the effects of low and high temperatures on M-CO_2_ battery components and their reaction mechanism, as well as the advancements made in extending operational ranges from room temperature to extremely low and high temperatures. It discusses strategies to enhance electrochemical performance at extreme temperatures and outlines opportunities, challenges, and future directions for the development of M-CO_2_ batteries.

## Highlights

- This review provides a comprehensive overview of the current research progress on metal–carbon dioxide (M-CO_2_) batteries across a broad temperature range (from room temperature to low/high temperatures).
- The challenges encountered by M-CO_2_ batteries under extreme low- and high-temperature conditions thoroughly discussed, along with strategies to address these challenges.
- The potential application scenarios and future directions of M-CO_2_ batteries across a broad temperature range are highlighted.

## Graphical abstract

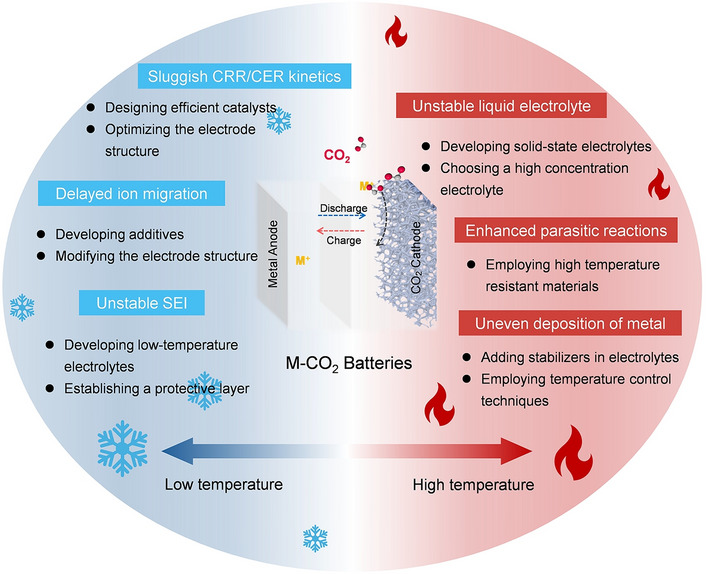

## Introduction

In recent years, the excessive consumption of non-renewable fossil fuels has raised global concerns regarding energy shortages, while a series of ecological and environmental issues resulting from excess CO_2_ emissions have also posed significant threats to human survival [1–4]. There is an urgent need to vigorously advance CO_2_-related technologies to achieve net-zero CO_2_ emissions. The metal–carbon dioxide (M-CO_2_, M = Li, Na, K, Al, Mg, Zn, etc.) battery, as an emerging energy storage device, enables direct the utilization of CO_2_ to mitigate its accumulation, rendering it a crucial component within the renewable energy network by virtue of its high-energy density [5–7]. For example, the energy density of the non-aqueous Li/Na-CO_2_ batteries can reach 1876 and 1136 Wh kg^−1^ based on the reversible reactions of 4Li/Na + 3CO_2_ ↔ 2Li_2_CO_3_/Na_2_CO_3_ + C [8–11]. In contrast, aqueous Zn/Al-CO_2_ batteries show a slightly lower energy density but can produce valuable carbon-containing chemicals such as CO, CH_4_, and C_2_H_4_ [12–14]. Therefore, M-CO_2_ batteries hold promising prospects for application in CO_2_ capture and utilization, energy conversion, and storage. The unique advantages of M-CO_2_ batteries will be particularly evident in high-concentration CO_2_ scenarios in the future, such as seabed exploration and undersea resource exploration [15–17].

**Fig. 1:**
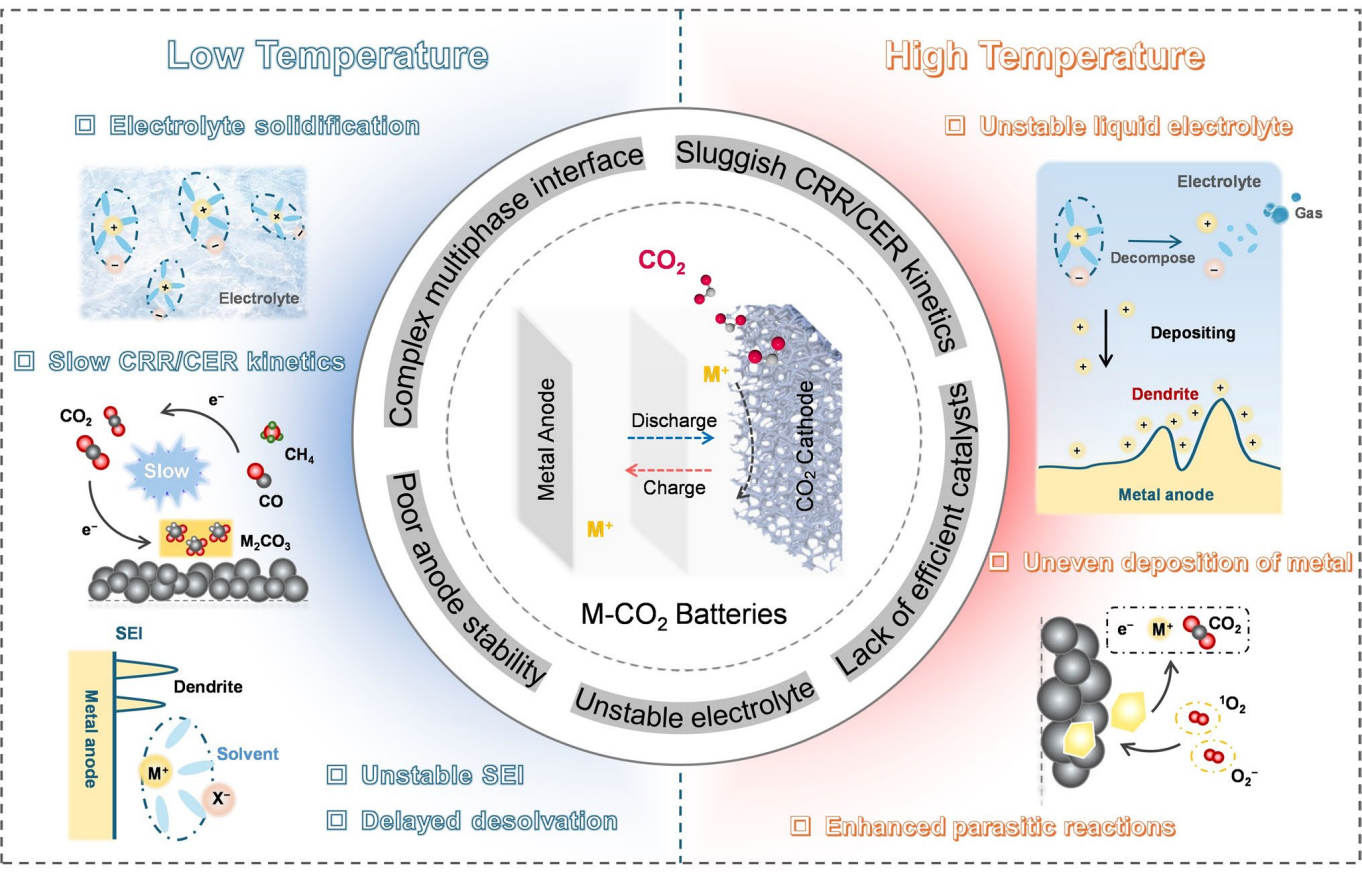
Challenges of wide-range temperature M-CO_2_ batteries

The electrochemical performance of M-CO_2_ batteries at room temperature (RT) has been the focus of extensive research efforts, resulting in significant advancements [18–22]. However, several formidable challenges still need to be addressed, including high overpotential during the discharge (CO_2_ reduction reaction, CRR)/charge (CO_2_ evolution reaction, CER) process, poor charge reversibility, and cycling capacity decay [23–25]. The reasons for these problems are multifaceted (Fig. 1): (i) the complex gas–liquid–solid multiphase reaction interface; (ii) the sluggish oxidation kinetics of carbonate products in non-protonic systems, coupled with the limited selectivity of reduction products in aqueous systems; (iii) the lack of efficient and stable cathode bifunctional catalysts; (iv) the growth of metal dendrites and the tendency of metal to be slowly corroded during the reaction process; (v) the lack of sufficiently stable electrolyte. Furthermore, it is noteworthy that the potential future applications of M-CO_2_ batteries necessitate their ability to maintain optimal operational performance even in extreme conditions such as low temperatures (LT) or high temperatures (HT) [26, 27]. The average temperature on the surface of Mars, for instance, is − 60 °C. Local temperatures in underground exploration scenes can surpass 200 °C, and the operational temperature requirement for energy storage batteries used in rockets and spaceplanes can reach 250 °C or higher [28, 29]. However, limited attention has been given by researchers to M-CO_2_ batteries that can operate efficiently across a wide range of temperatures.

As shown in Fig. 1, the operation of M-CO_2_ batteries at extremely low or high environmental temperatures remains significant challenges. At LT, the primary obstacle lies in the sluggish kinetic behavior. The mass transfer of metal ions (M^+^) and CO_2_ is limited, resulting in reduced reaction rates at both the cathode and anode, increased overpotential, and a shortened battery lifespan. Additionally, when the temperature drops to the freezing point of the electrolyte, the electrochemical window significantly narrows. On the contrary, at elevated temperatures, the reaction kinetics of M-CO_2_ batteries accelerate. In 2013, Archer's group pioneered the development of a Li-CO_2_ battery and conducted an extensive investigation into its performance at temperatures ranging from 60 to 100 °C [30]. It was found that temperature influences the thickness and structure of the insulating product, with higher temperatures enhancing discharge potential and battery capacity. However, elevated temperatures also pose challenges such as increased reactivity of the metal anode, dendrite growth, electrolyte evaporation, and side reactions at the anode–electrolyte interface.

In recent years, both domestic and international research progress on metal-CO_2_ batteries has undergone a comprehensive review, including the key reaction mechanisms, the design of efficient cathode catalysts, and anode protection [5, 6]. There are also some reviews related to lithium-ion batteries, sodium-ion batteries, and zinc-ion batteries that exhibit excellent temperature adaptability [31–34]. However, there has been no comprehensive review on M-CO_2_ batteries in a wide-temperature range. When exposed to extreme conditions, M-CO_2_ batteries encounter distinct challenges compared to metal-ion batteries. These differences primarily encompass the following aspects: (i) The behavior of gaseous CO_2_ diffusion, solubility, and reactivity with the electrode varies across different temperatures. (ii) Maintaining stability at extreme temperatures is difficult for the gas–liquid–solid three-phase interface. (iii) It is challenging for catalysts to maintain high activity over a wide-temperature range. (iv) Metal deposition/stripping processes are influenced by potential side effects arising from CO_2_ reduction. (v) The electrolyte in M-CO_2_ batteries not only requires effective ion conduction but also active participation in the CO_2_ reduction process, potentially leading to undesirable side reactions. Herein, this paper reviews relevant research progress of M-CO_2_ batteries operated from RT to extremely low and extremely HT. It provides an elaborate discussion on the effects of low and HT on battery components while summarizing effective strategies for enhancing battery's electrochemical performance at high and low temperatures. Finally, it proposes opportunities, challenges, and future development directions for M-CO_2_ batteries.

## Operate Across RT to LT

Lowering the ambient temperature exacerbates several issues in secondary batteries, and M-CO_2_ batteries are no exception [35–39]. These issues specifically include: (i) Reduced conductivity of the electrolyte, which limits the transport efficiency of M^+^ and CO_2_ during the reaction process. (ii) Slower electrode reaction kinetics, encompassing metal deposition and stripping on the anode side, as well as CO_2_ reduction and evolution on the cathode side. (iii) Weakened desolvation effects at the electrode/electrolyte interface. (iv) Potential phase transitions or structural fractures in numerous solid-phase catalysts, leading to diminished catalytic activity [40–43]. As a result, higher energy input is often needed to propel the charge–discharge process, directly resulting in elevated overpotential and shortened cycle life. To enhance their reaction kinetics and cycle stability, various research groups have prioritized different approaches for distinct systems, including non-aqueous, aqueous, and solid-state M-CO_2_ batteries. The following sections will provide a detailed overview of the research advancements in these diverse systems, emphasizing their improvement strategies and performance under low-temperature conditions.

### Non-Aqueous M-CO_2_ Batteries

**Fig. 2:**
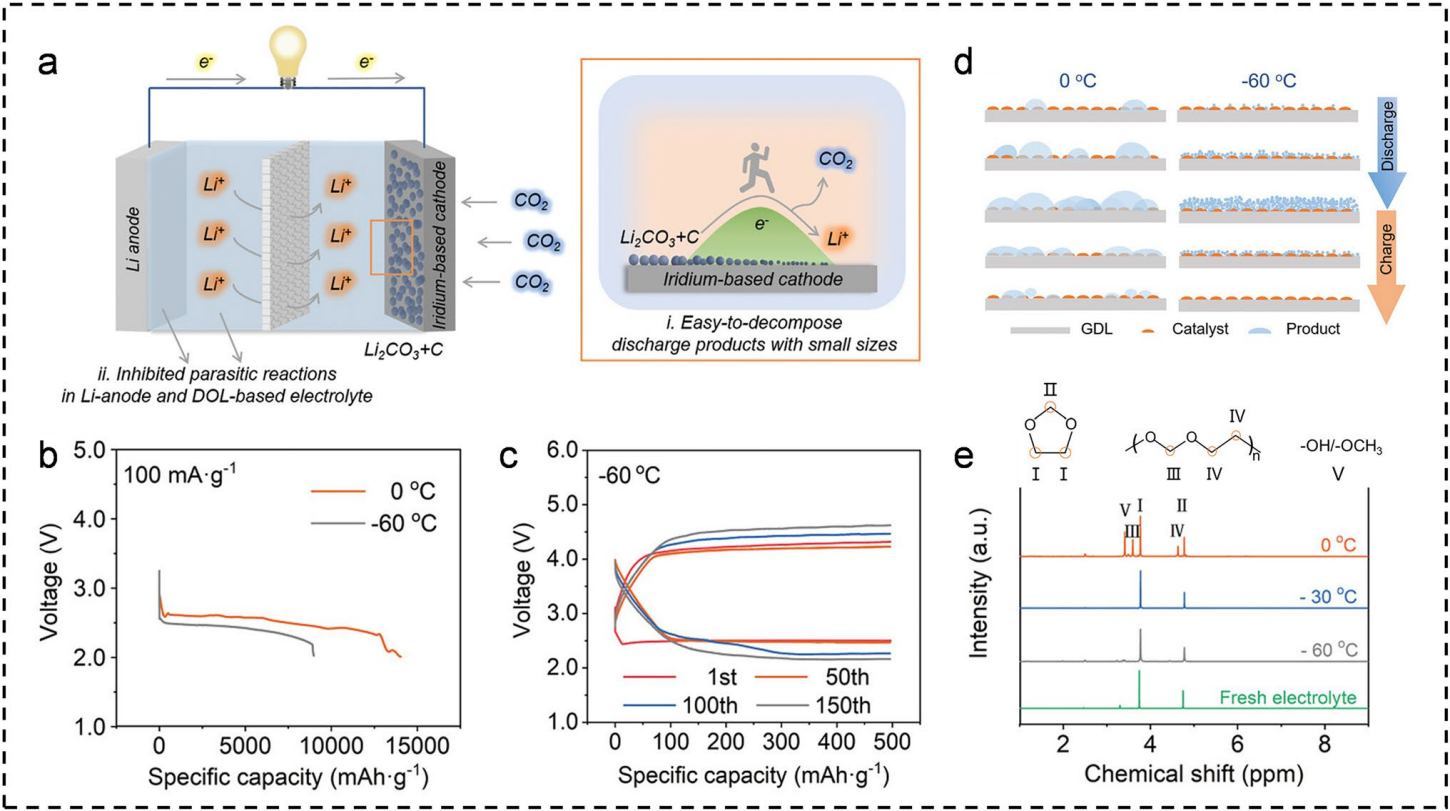
**a** Schematic illustration to Li–CO_2_ batteries with DOL-based electrolyte and Ir-based catalysts in ultra-low-temperature environments. **b** Deep discharge profiles. **c** Selected discharge–charge curves at 100 mA g^−1^ and − 60 °C. **d** A schematic diagram depicting the influence of temperature on the formation and decomposition of products. **e** ^1^H-NMR spectra of the fresh and cycled electrolytes after ten cycles at 0, − 30, and − 60 °C

In the early development of non-aqueous M-CO_2_ batteries, Peng’s group designed and assembled a Swagelok-type Li–CO_2_ battery that exhibited exceptional electrochemical performance even at extremely LT (− 60 °C) [44]. As depicted in Fig. 2a, they used Li metal as the anode, a lithium bis (trifluoromethylsulfonyl) imide (LiTFSI)-based 1,3-dioxolane (DOL) as the electrolyte, and a gas diffusion electrode (GDL) coated with an iridium catalyst as the cathode. This DOL-based electrolyte has a low freezing point (− 100 °C), high ionic conductivity (2.26 mS cm^−1^ at − 80 °C), and excellent electrochemical stability. The introduction of DOL effectively suppresses electrolyte side reactions, while the Ir-coated GDL ensures high catalytic activity for both the CRR and CER. Ultimately, the battery exhibited a remarkable depth-of-discharge capacity of 8,976 mA g^−1^ and an exceptional cycle life of 150 cycles (equivalent to 1,500 h) at an ultra-low temperature of − 60 °C and a current density of 100 mA g^−1^ (Fig. 2b, c). It is noteworthy that the cycling performance of the Li–CO_2_ battery at − 60 °C surpasses that at 0 and − 30 °C. This is because, at − 60 °C, the slower growth of products leads to the formation of small, more easily reversible particulate products (Fig. 2d). In addition, the ^1^H-nuclear magnetic resonance (^1^H-NMR) results in Fig. 2e confirm that the low-temperature environment can effectively suppress side reactions associated with the DOL electrolyte, such as DOL molecule decomposition and ring-opening polymerization, and mitigate side reactions caused by inevitable moisture in the system. Therefore, while low-temperature environments do not positively enhance the main battery reactions, they also mitigate the occurrence of side reactions. Similarly, researchers are investigating various solvents to enhance electrolyte performance. Kang et al. selected N,N-dimethylacetamide (DMAc) with high ionic conductivity as the solvent for the electrolyte in the CO_2_-assisted Li-O_2_ battery [45]. Mechanistically, batteries based on the traditional electrolyte (LiTFSI in tetraethylene glycol dimethyl ether, LiTFSI/TEGDME) generate anhydride-linked C_2_O_6_^2−^ products during discharge, whereas those employing LiNO_3_/DMAc produce peroxo-linked C_2_O_6_^2−^. Compared to the anhydride-linked C_2_O_6_^2−^, the peroxo-linked C_2_O_6_^2−^ exhibits enhanced stability, leading to significant enhancements in cycling performance (100 cycles at − 15 °C). They pointed out that the dielectric constant and Gutman donor count of the non-proton solvent can influence the molecular structure of C_2_O_6_^2−^. Therefore, developing novel organic electrolyte systems could potentially improve low-temperature electrochemical performance by altering the battery’s reaction pathways.

**Fig. 3:**
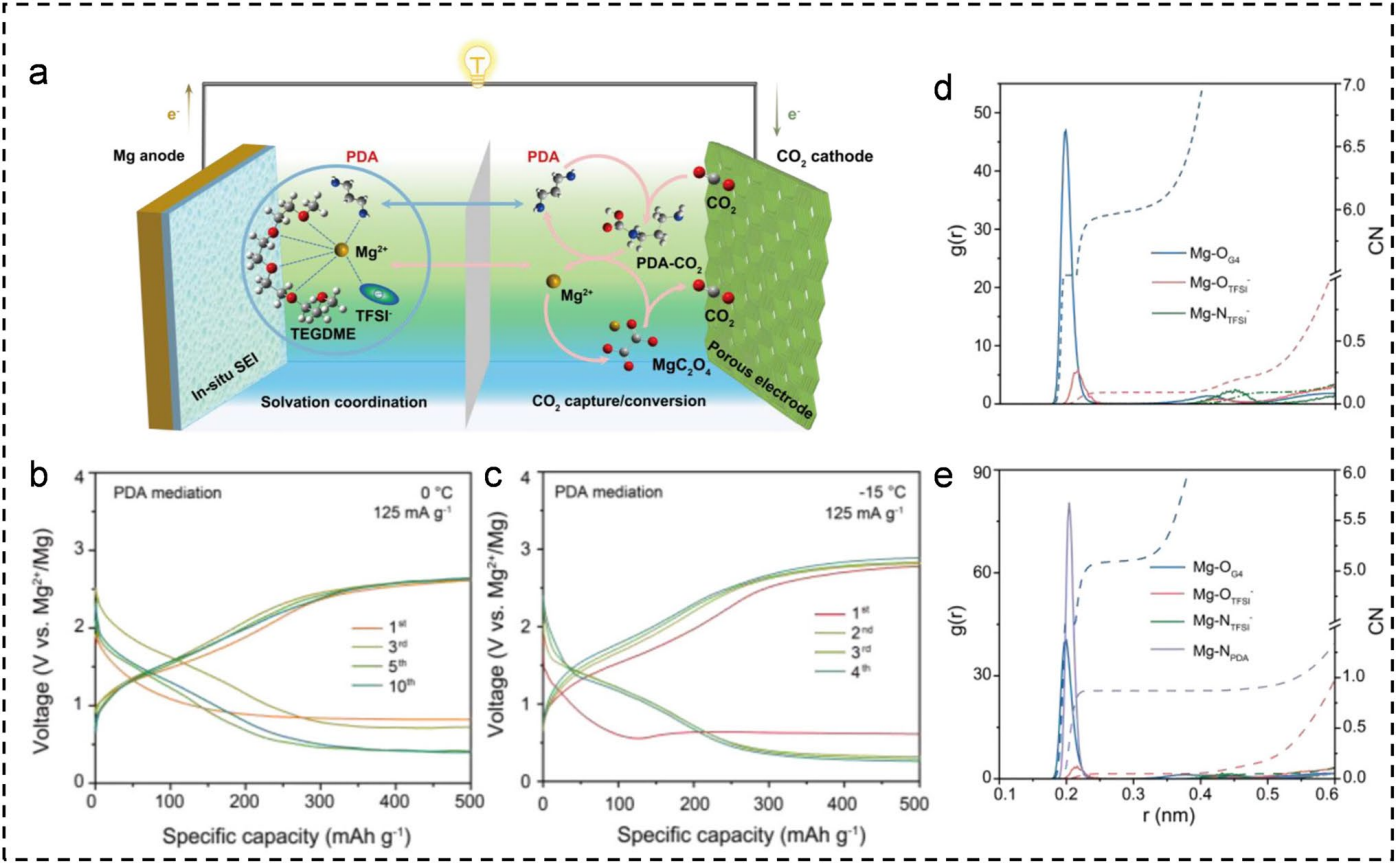
**a** Schematic diagram of a Mg-CO_2_ battery utilizing PDA additive as a dual-function medium. Selected discharge/charge cycles at **b** 0 °C and **c** − 15 °C at 125 mA g^−1^. The RDF and CN of TFSI^−^, G4 and PDA in **d** baseline and **e** PDA-mediated electrolytes

Additionally, Guo et al. successfully constructed a highly reversible Mg–CO_2_ battery mediated by liquid 1, 3-propylene amine (PDA) [46]. The introduction of PDA facilitates the chemical adsorption of CO_2_ and modulates the solvent coordination of Mg^2+^, thereby conferring benefits to both the anode and cathode. As shown in Fig. 3a, PDA enhances the compatibility of the multiphase interface and facilitates rapid desolvation and diffusion of Mg^2+^ to form MgC_2_O_4_ that is easily decomposed reversibly, thus significantly improving the CRR/CER process on the cathode. On the other hand, PDA promotes the in situ generation of a solid electrolyte interface (SEI) primarily composed of organic compounds at the anode, enabling efficient conduction of Mg^2+^ ions to ensure high reversibility during Mg deposition/dissolution. Therefore, PDA-mediated Mg–CO_2_ batteries achieve stable cycling performance for up to 70 cycles at 200 mA g^−1^ and exhibit excellent rate performance with an overpotential of less than 1.5 V in the range of 100–2000 mA g^−1^. Notably, the battery functions normally at temperatures as low as 0 or − 15 °C (Fig. 3b, c), which can be related to the robust interaction between the nitrogen atoms in the PDA (N_PDA_) with a strong electronegativity and Mg^2+^ that effectively mitigates the constraints between TEGDME (G4) and Mg^2+^. This hypothesis is supported by the radial distribution function (RDF) and coordination number (CN) results of anions or solvents obtained from molecular dynamics simulations (Fig. 3d, e). The addition of PDA reduces the CN value of Mg–O_G4_ from 6 to 5 and introduces Mg-N_PDA_ coordination.

**Fig. 4:**
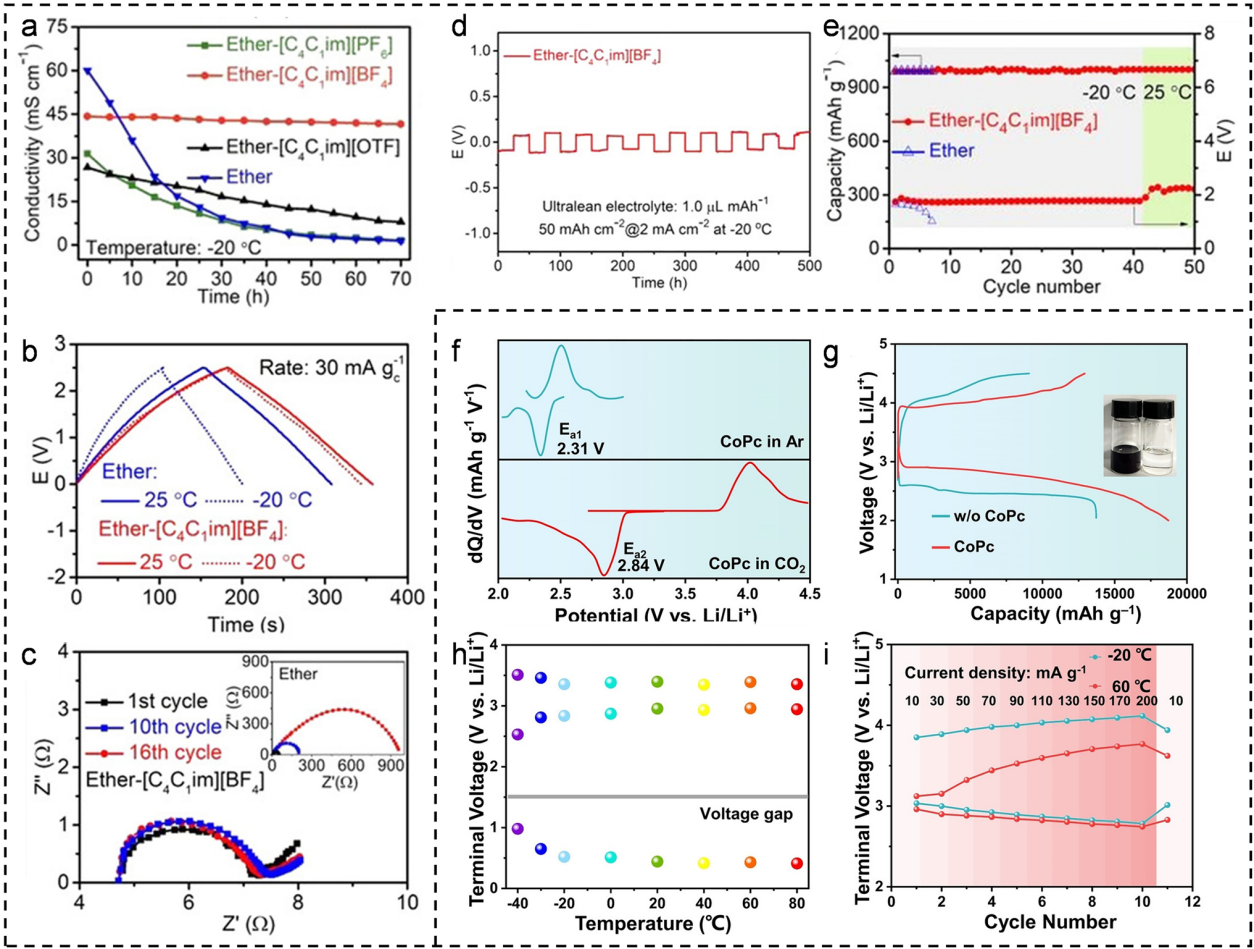
**a** The rate capability of Na symmetric batteries with different electrolytes. **b** Charge–discharge curves of the double layer capacitor. **c** EIS of symmetric cells after different cycles at − 20 °C. **d** Voltage–time curve of a Na symmetric cell. **e** Cycling performance of the Na-CO_2_ batteries with ether-[C_4_C_1_im][BF_4_] electrolyte from − 20 to 25 °C. **f** dQ/dV curves and **g** full discharge–charge curves of Li-CO_2_ batteries. **h** Median voltage and voltage gap of Li-CO_2_ battery with CoPc across a wide-temperature range. **i** Rate performance working at − 20 and 60 °C

For the challenges of low plating/stripping capacity and limited cycling stability of a metal Na anode at LT, Hu’s group designed an ionic liquid-based composite electrolyte, 1 M NaPF_6_ in a mixture of 1-butyl-3-methylimidazolium tetrafluoroborate and diglyme (denoted as ether-[C_4_C_1_im][BF_4_]) [47]. This electrolyte demonstrates high ionic conductivity (42 mS cm^−1^ at − 20 °C) and exceptional solvent dehydration capability at LT (Fig. 4a, b). Additionally, the electrochemical impedance spectroscopy (EIS, Fig. 4c) before and after cycling indicates that a more stable SEI film has formed in the battery containing the ether-[C_4_C_1_im][BF_4_]. As depicted in Fig. 4d, the Na metal battery demonstrated a high reversible capacity of 50 mAh cm^−2^ over 500 h in the lean electrolyte (1.0 μL mAh^−1^). The Na–CO_2_ battery based on the composite electrolyte can be stably cycled for 50 cycles under conditions ranging from − 20 to 25 °C (Fig. 4e). Recently, the research group employed cobalt phthalocyanine (CoPc) as a homogeneous catalyst and incorporated it into the ether-based electrolyte to elevate the discharge potential of the Li–CO_2_ battery to 2.98 V (the theoretical value is 2.8 V) and enhance the discharge capacity to 18,724 mAh g^−1^ (Fig. 4f, g) [48]. Electrochemical analysis and density functional theory (DFT) calculations reveal that CoPc adsorbs CO_2_ to form a discharge intermediate product, C_33_H_16_CoN_8_O_2_, during the resting state. Subsequently, the C_33_H_16_CoN_8_O_2_ intermediate gains electrons and reacts with Li^+^ to produce Li_2_CO_3_ and C, while releasing CoPc. The discharge voltage of the Li–CO_2_ battery is directly correlated with the reduction potential of the intermediate, resulting in a voltage that exceeds the theoretical value. Furthermore, the battery with CoPc exhibits marked cycling performance, capable of stable cycling for 1600 h at a current density of 100 mA g^−1^ (with a cutoff capacity of 1000 mAh g^−1^). Notably, the freezing point of the LiTFSI/TEGDME electrolyte with 50 mM CoPc can reach − 36.6 °C. Although the voltage gap at − 30 °C (0.65 V) is slightly higher than at other temperatures, the discharge voltage plateau remains at 2.8 V. Additionally, the Li–CO_2_ battery with CoPc shows viable cycling performance at both − 20 and 60 °C (Fig. 4h, i), underscoring its exceptional temperature adaptability.

The current research on non-aqueous M-CO_2_ batteries in low-temperature environments has achieved some progress. The primary focus of the research encompasses the design and optimization of electrolyte systems, catalyst selection, enhancement of interface stability, and mitigation of side reactions by adding functional additives. Specifically, the development of electrolytes characterized by low freezing points, high ionic conductivity, and robust stability (such as DOL and DMAc) has emerged as a crucial strategy for enhancing performance at reduced temperatures. Concurrently, the incorporation of efficient catalysts such as Ir and CoPc can markedly enhance the kinetics associated with CRR and CER, thereby extending battery cycle life. Furthermore, researchers are dedicated to improving overall battery performance via interface regulation strategies aimed at optimizing charge transport and metal deposition processes at three-phase interfaces. However, despite notable breakthroughs in inhibiting side reactions and enhancing electrochemical properties under low-temperature conditions, several critical issues have been overlooked. For instance, the long-term stability of catalysts along with their structural transformations at extremely LT remains inadequately explored. As temperature decreases significantly affects CO_2_ gas phase transport dynamics and reactivity, thus the long-term cycles of overall battery warrant further investigation.

### Aqueous M-CO_2_ Batteries

Similar to organic electrolytes, aqueous electrolytes face significant challenges at LT, including a notable reduction in ion mobility and slow reaction kinetics at the interface, which can result in electrode material passivation or the formation of unstable interface layers, severely affecting the electrochemical performance of aqueous batteries [49–52]. More critically, water freezes at 0 °C, directly causing the electrolyte to lose its fluidity and ion conductivity [53, 54]. Researchers have proposed various strategies to improve the low-temperature performance of aqueous electrolytes: (i) Using low-temperature antifreeze additives: Adding antifreeze additives such as ethylene glycol, glycerol, and dimethyl sulfoxide (DMSO) can lower the freezing point of the electrolyte, maintaining its fluidity and ion conductivity at LT. (ii) Optimizing electrolyte composition: Optimizing salt concentration and selecting suitable salts can enhance the electrolyte’s performance. For example, adding high concentrations of lithium salts (like LiTFSI) can improve the electrolyte's conductivity and low-temperature stability. (iii) Developing novel gel electrolytes: Creating new gel electrolytes that exhibit better mechanical properties and electrochemical stability at LT. (iv) Surface modification of electrodes: Modifying the electrode surface to form a stable SEI. For instance, coating the electrode with a conductive polymer or inorganic material can effectively mitigate interface issues at LT [55–59]. However, research on improving the low-temperature performance of aqueous M-CO_2_ batteries is still relatively scarce.

**Fig. 5:**
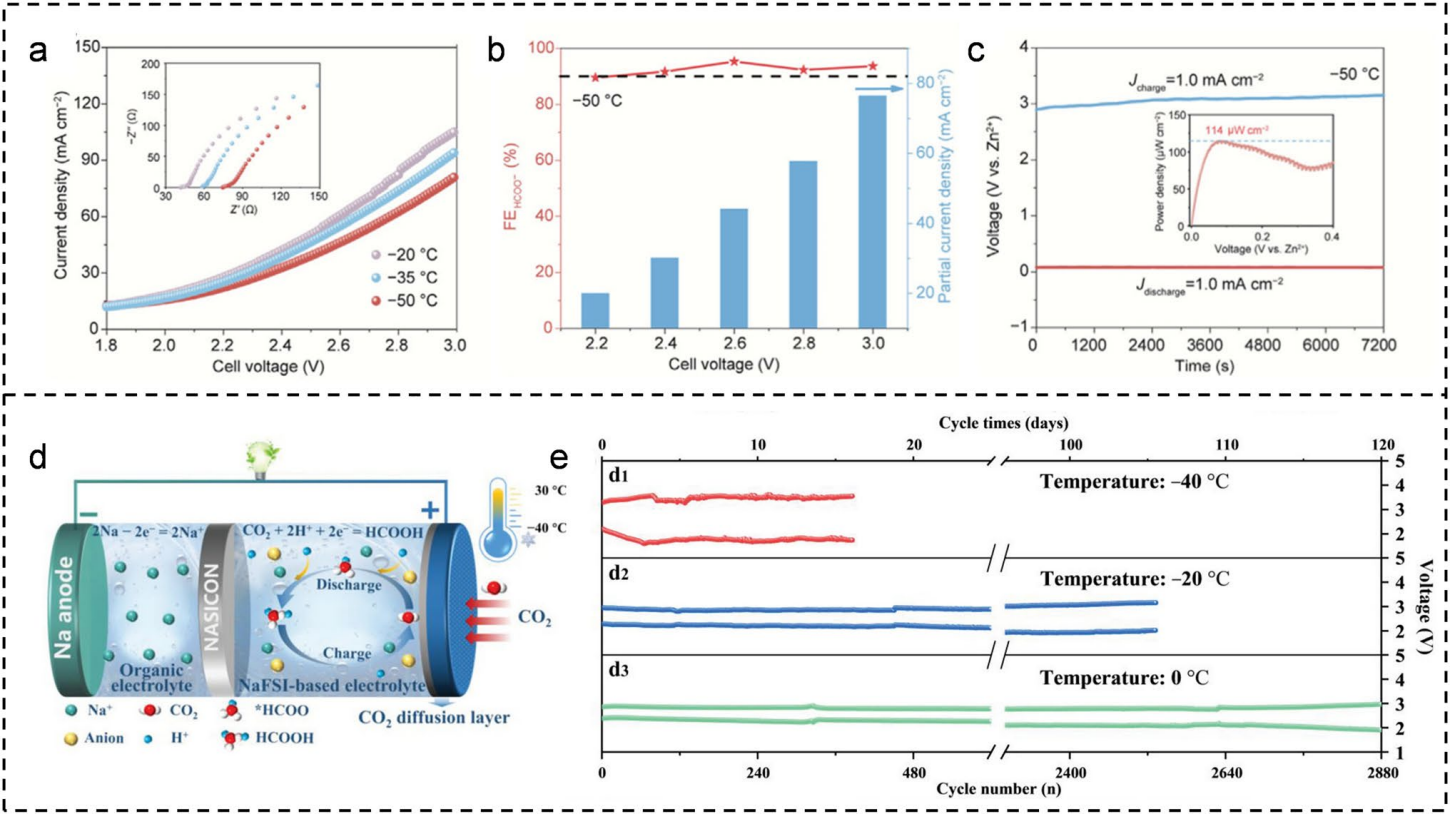
**a** LSV curves of Bi_5_O_7_I in 3 mol L^−1^ CsOH (DMSO) at different temperature. Inset: EIS curves. **b** FE_HCOO_ and partial current density at − 50 °C. **c** The galvanostatic discharge–charge curves of Zn-CO_2_ batteries. Inset: the power density. **d** Schematic diagram of hybrid Na-CO_2_ battery based on NaFSI electrolytes. **e** Cycling curves at different temperatures (40, 20, and 0 °C)

For example, Lei et al. synthesized the Bi_5_O_7_I catalyst by a one-step ultrasonic reduction method [55]. The faraday efficiency of formate salts (FE_HCOO_^−^) can achieve over 90% (a maximum value of 96.14% at − 0.95 V) in the assembled H-type electrolyzer with this catalyst as the anode at 20 °C. Even when the working temperature drops to − 5 °C, the FE_HCOO-_ remains above 90%. However, the initial 1 mol L^−1^ KOH electrolyte starts to solidify as the temperature decreases from − 5 to − 20 °C. Although the enhancement of alkali concentration can enhance the low-temperature adaptability of the electrolyte, may adversely affect solution viscosity and solvation, thereby impeding gas diffusion and mass transfer. Therefore, they developed an electrolyte formulation comprising 40% DMSO and 60% CsOH with a concentration of 3 mol L^−1^, enabling operation at temperatures as low as − 50 °C without freezing. As depicted in Fig. 5a, the electrical impedance increases gradually as the temperature drops from − 20 to − 50 °C, resulting in a corresponding decrease in current density. The FE_HCOO-_ remained consistently above 90% even at a temperature as low as − 50 °C, while achieving a remarkable current density of up to 76 mA cm^−2^ at 3.0 V, thus demonstrating the practicality of CO_2_ reduction under extremely cold conditions (Fig. 5b). The Zn–CO_2_ battery, assembled with Bi_5_O_7_I as the cathode and a zinc plate as the anode, shows exceptional rate performance (1, 2, and 3 mA cm^−2^), high peak power density (114 µW cm^−2^), and remarkable stability (14 h at 1 mA cm^−2^) even at − 50 °C (Fig. 5c).

Besides, Liang et al. proposed the utilization of a salt-in-water electrolyte to regulate the CO_2_ reduction pathway by establishing an optimal reaction environment in the Na–CO_2_ hybrid battery (Fig. 5d) [60]. DFT calculations and experimental results demonstrated that by employing a sodium bis(fluorosulfonyl)imide (NaFSI)-based salt-in-water electrolyte as the cathode solution, it is possible to effectively control the relative concentration of H/O atoms at the interface between the electrolyte and catalyst, thereby modulating the CO_2_ reduction pathway to yield formic acid not traditional solid product Na_2_CO_3_. Ultimately, a hybrid Na–CO_2_ battery based on a 27 M NaFSI + 8 M NaClO_4_ (Na(FSI)_27_(ClO_4_)_8_) electrolyte showed an ultra-high specific discharge capacity of 148.1 mAh cm^−2^ along with excellent cycling performance observed over 1200 cycles at 30 °C. Furthermore, as depicted in Fig. 5e, stable cycling performance up to 2534 times at − 20 °C could be maintained due to its highly reversible liquid product HCOOH and outstanding low-temperature properties exhibited by this electrolyte.

Researchers have made initial progress in the domain of low-temperature aqueous M-CO_2_ batteries, mainly focusing on improving their electrochemical performance at LT through the optimization of electrolyte systems and modifications to electrode surfaces. These efforts encompass the addition of antifreeze, optimization of salt concentrations, development of novel gel electrolytes, and modifications to the electrode surface. However, there are still some problems that have not been fully explored. For instance, how to further reduce the freezing point of the electrolyte while ensuring the long-term stability of the electrode interface and reaction reversibility. And it is imperative to focus not only on the electrolyte itself but also on optimizing its interfacial coupling with the electrodes.

### Solid-State M-CO_2_ Batteries

**Fig. 6:**
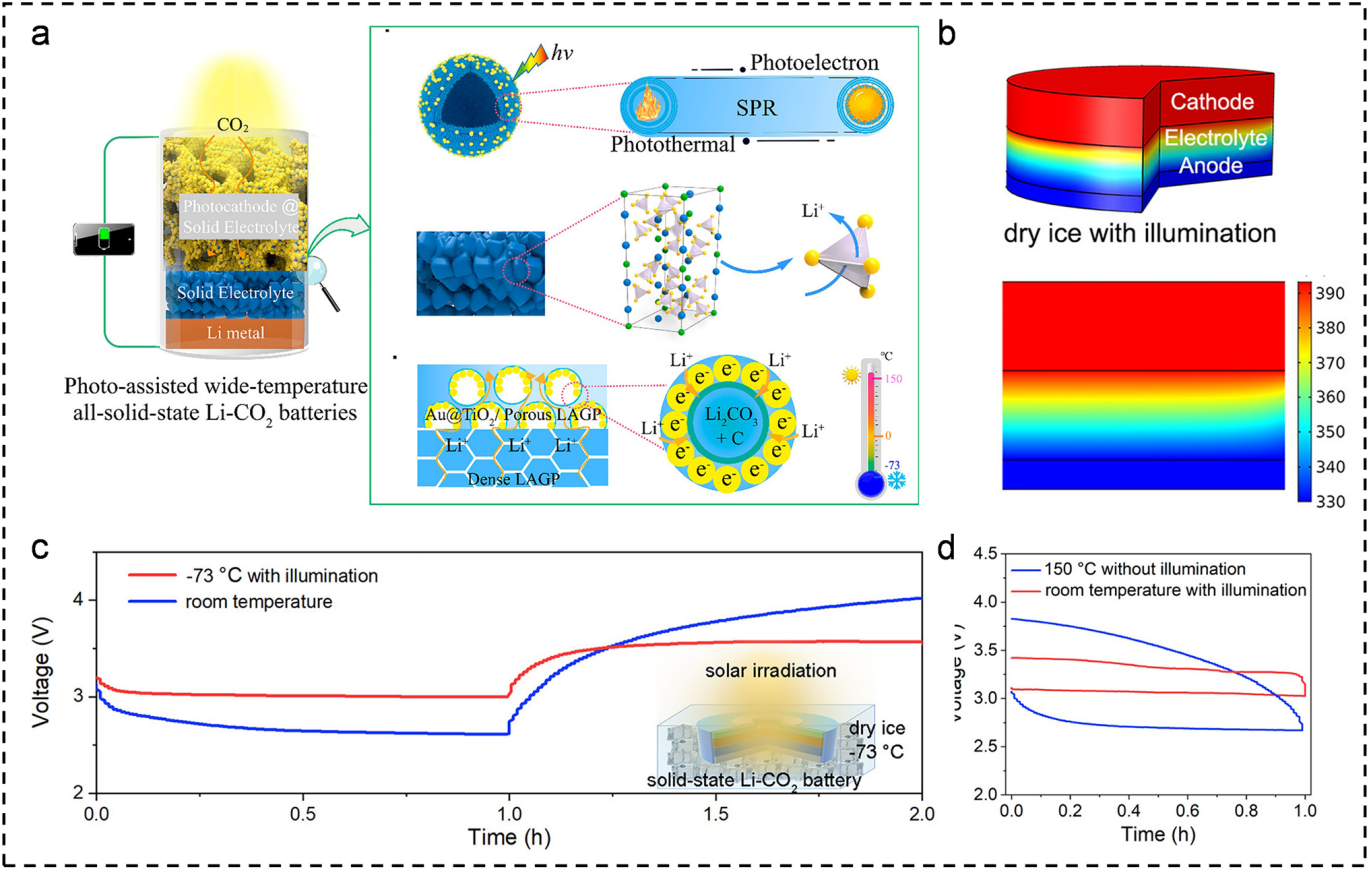
**a** The schematic diagram of a solar photothermal all-solid-state Li-CO_2_ battery with an integrated structure. **b** Temperature distribution at − 73 °C with illumination. **c** First discharge–charge curves at RT and − 73 °C with illumination. **d** First discharge–charge curves at RT with illumination and ~ 150 °C without illumination

The solid electrolyte itself exhibits a relatively high interfacial resistance at RT, let alone at lower temperatures. With significant advancements in solid-state electrolytes, all-solid-state batteries now demonstrate excellent electrochemical performance at both room and elevated temperatures [61, 62]. However, there are still substantial challenges associated with operating below 0 °C. Recently, Xu's research group has made a breakthrough by designing and constructing a novel photo-assisted all-solid-state Li-CO_2_ battery that utilizes the photothermal effect to enable safe, stable, and efficient operation across a wide-temperature range (Fig. 6a) [63]. In this battery design, a super-thin layer of Li_1.5_Al_0.5_Ge_1.5_(PO_4_)_3_ (LAGP) solid electrolyte is applied onto the Li anode, while the Au@TiO_2_ photocatalyst is loaded into porous LAGP structure. The all-solid-state Li-CO_2_ battery with the Au@TiO_2_/LAGP/LAGP (ATLL) dual-layer framework demonstrated an exceptionally low polarization of 0.25 V under illumination, along with a stable cycling life of 400 h and a high round-trip efficiency of 92.4%. The COMSOL simulation results in Fig. 6b demonstrate that, even at an ultra-low temperature of − 73 °C, the cathode temperature can reach approximately 113 °C under light illumination. Additionally, the surface temperature of anode can also reach up to 63 °C based on the efficient heat conduction facilitated by the LAGP framework. Ultimately, the battery was able to attain a remarkably low polarization potential of 0.6 V even at − 73 °C owing to the exceptional photothermal conversion capability of Au@TiO_2_ (Fig. 6c). Furthermore, the overpotential of the all-solid-state Li-CO_2_ battery at 150 °C was 0.8 V without illumination, higher than the overpotential under RT with illumination, indicating the crucial role of light in this system (Fig. 6d). The author concludes that the battery exhibits exceptional electrochemical performance across a wide-temperature range (− 73 to 150 °C), which can be attributed to three key factors: (i) The photo-induced charge carriers on the Au@TiO_2_ heterostructure can directly enhance the kinetics of CRR/CER, while the photothermal effect enables continuous self-heating of the battery, facilitating diffusion and transfer of CO_2_ and Li^+^ at LT. (ii) The NASICON-structured LAGP electrolytes feature outstanding electrical conductivity and thermal stability. (iii) The porous integrated LAGP cathode coated with Au@TiO_2_ can expose more three-phase interfaces, promoting efficient transfer of Li^+^ and heat. This innovative approach to photothermally assisted battery design opens up new possibilities for improving the electrochemical performance of solid-state M-CO_2_ batteries over a wide-temperature range. While the photothermal effect presents a promising avenue for temperature regulation, its reliance on external energy sources constrains its practical applicability in non-laboratory environments. Therefore, addressing the intrinsic limitations of solid electrolytes regarding ionic conductivity and interfacial compatibility, particularly at ultra-low temperatures, remains paramount.

## Operate Across RT to HT

Increasing temperature can significantly accelerate the reaction kinetics in M-CO_2_ batteries. Huang group utilized in situ environmental transmission electron microscopy (ETEM) to investigate the electrochemical properties of Li_2_CO_3_ in Li–CO_2_ batteries during discharge and charge processes [64]. The results showed that the Li_2_CO_3_ layer formed on the cathode is difficult to decompose at RT but rapidly decomposes at higher temperatures. Therefore, the combination of HT and applied voltage can promote the rapid decomposition of carbonate products. However, this also introduces several issues. In particular, M-CO_2_ batteries with liquid electrolytes will encounter several significant challenges. The primary concerns are as follows: (i) Liquid electrolytes may experience thermal decomposition, volatilization, or degradation, especially those organic solvents; (ii) although elevated temperatures can enhance the ionic conductivity of the electrolyte, they may also accelerate reactions within the electrolyte, resulting in an increase in side reactions; (iii) the electrolyte interface reaction becomes more pronounced, characterized by the non-uniform deposition of the metal anode and the dissolution of the catalyst. These issues directly lead to reduced stability, shorter lifespan, and even uncontrolled internal reactions and damage, affecting the battery’s reliability and safety. Therefore, the feasibility of employing liquid electrolytes in M–CO_2_ batteries is primarily contingent upon the thermal stability, electrochemical stability, and interfacial stability of the electrolyte at elevated temperatures. By strategically designing heat-resistant electrolyte materials and optimizing both the electrode architecture and overall battery configuration, the potential for utilizing liquid electrolytes in high-temperature M–CO_2_ batteries remains promising. For instance, incorporating ionic liquids with high boiling points and excellent thermal stability, utilizing ultra-high boiling organic solvents, or introducing heat-resistant additives can be effective strategies. Additionally, modifying the electrode surface interface or applying coatings to enhance interfacial stability and developing a flow-type electrolyte system to efficiently manage by-products while preventing their accumulation are also viable approaches.

**Fig. 7:**
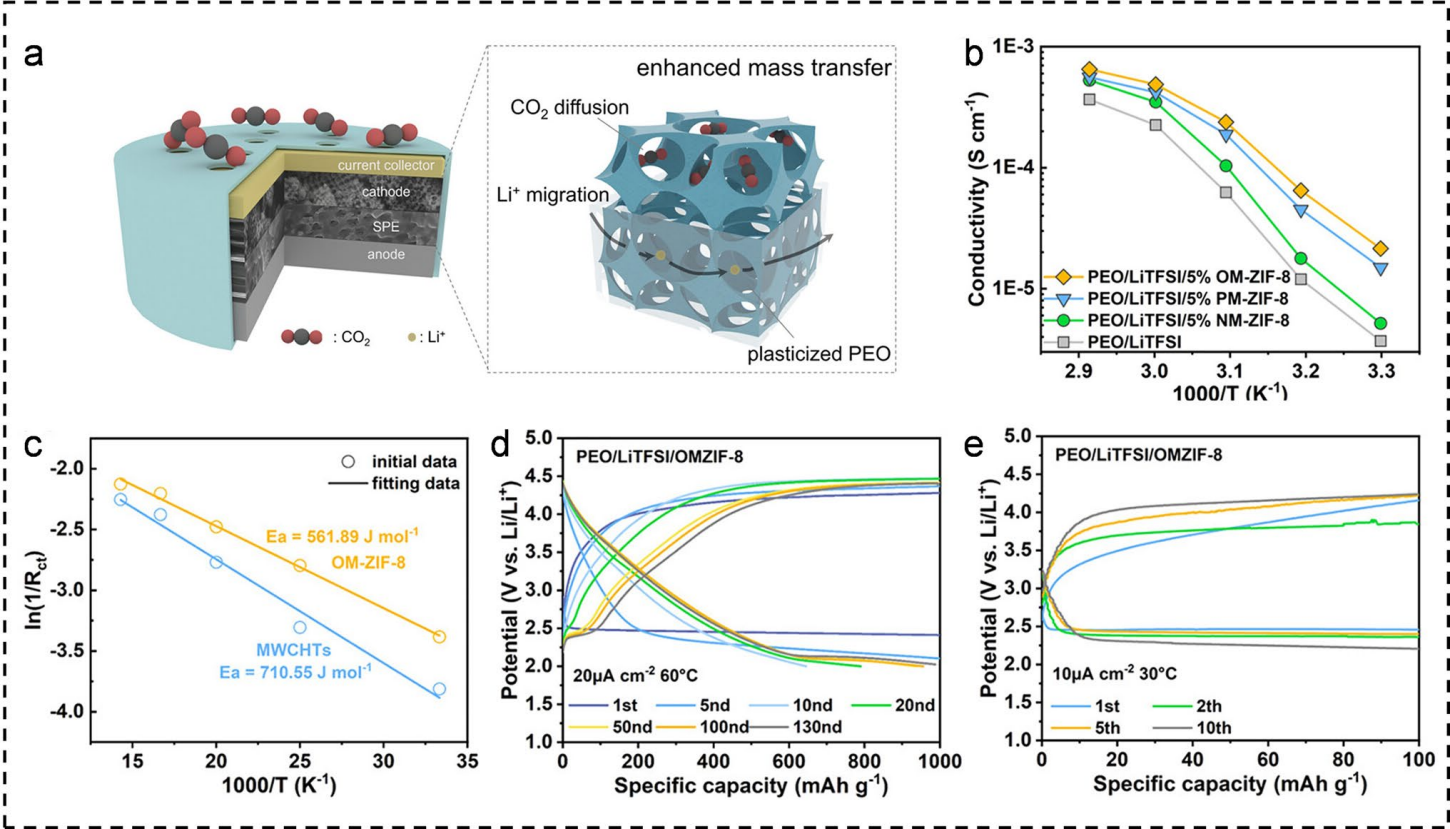
**a** Schematic diagram of a Li-CO_2_ battery with BCHPSs. **b** Temperature-dependent ionic conductivities for OM-ZIF-8/SPE. **c** E_a_ for CO_2_ adsorption. The selected discharge–charge curves of Li-CO_2_ batteries with OM-ZIF-8 at **d** 60 °C and **e** 30 °C

In comparison with liquid electrolytes, solid electrolytes offer significant advantages in the development of high-energy density and high-safety M-CO_2_ batteries with a wide operating temperature range due to their elevated Young’s modulus and stable structural properties across a broad temperature spectrum [7, 65–71]. The Hu research group has extensive experience in the design and construction of solid electrolytes, particularly in terms of advantageous features such as high ionic conductivity and stability, and has successfully achieved stable operation of the M-CO_2_ battery at HT [72–75]. As shown in Fig. 7a, they recently designed an ordered zeolitic imidazolate framework-8 containing macro-porous and microporous (OM-ZIF-8), which served as both an additive for solid polymer electrolyte (SPE) and the cathode in Li–CO_2_ batteries. The unique bicontinuous hierarchical porous structures (BCHPSs) can effectively promote mass transport in both the solid electrolyte and the cathode. Adding OM-ZIF-8 to the SPE (OM-ZIF-8/SPE) results in an ionic conductivity of 4.87 × 10^−4^ S cm^−1^ and a Li^+^ transference number of 0.642 at 30 °C. When the operating temperature is increased to 60 °C, the ionic transport capacity of OM-ZIF-8/SPE is further enhanced, while the activation energy for CO_2_ adsorption by the OM-ZIF-8 cathode is as high as 561.89 J mol^−1^ (Fig. 7b, c). Ultimately, the Li–CO_2_ battery assembled with OM-ZIF-8 exhibits excellent cycling stability and stable charge/discharge profiles at both RT and 60 °C (Fig. 7d, e). Specifically, at a working temperature of 60 °C and a current density of 20 μA cm^−2^, the battery can stably cycle 133 times (> 1000 h). The outstanding cycle performance is related to the rich Lewis acid sites on the BCHPSs of OM-ZIF-8, which can effectively promote the dissociation of lithium salt and the adsorption and conversion of CO_2_. Besides, the main cause of battery deactivation is the “dead Li_2_CO_3_” produced during the reaction process. The BCHPSs can effectively inhibit the formation of “dead Li_2_CO_3_” to extend the cycle life of the battery.

**Fig. 8:**
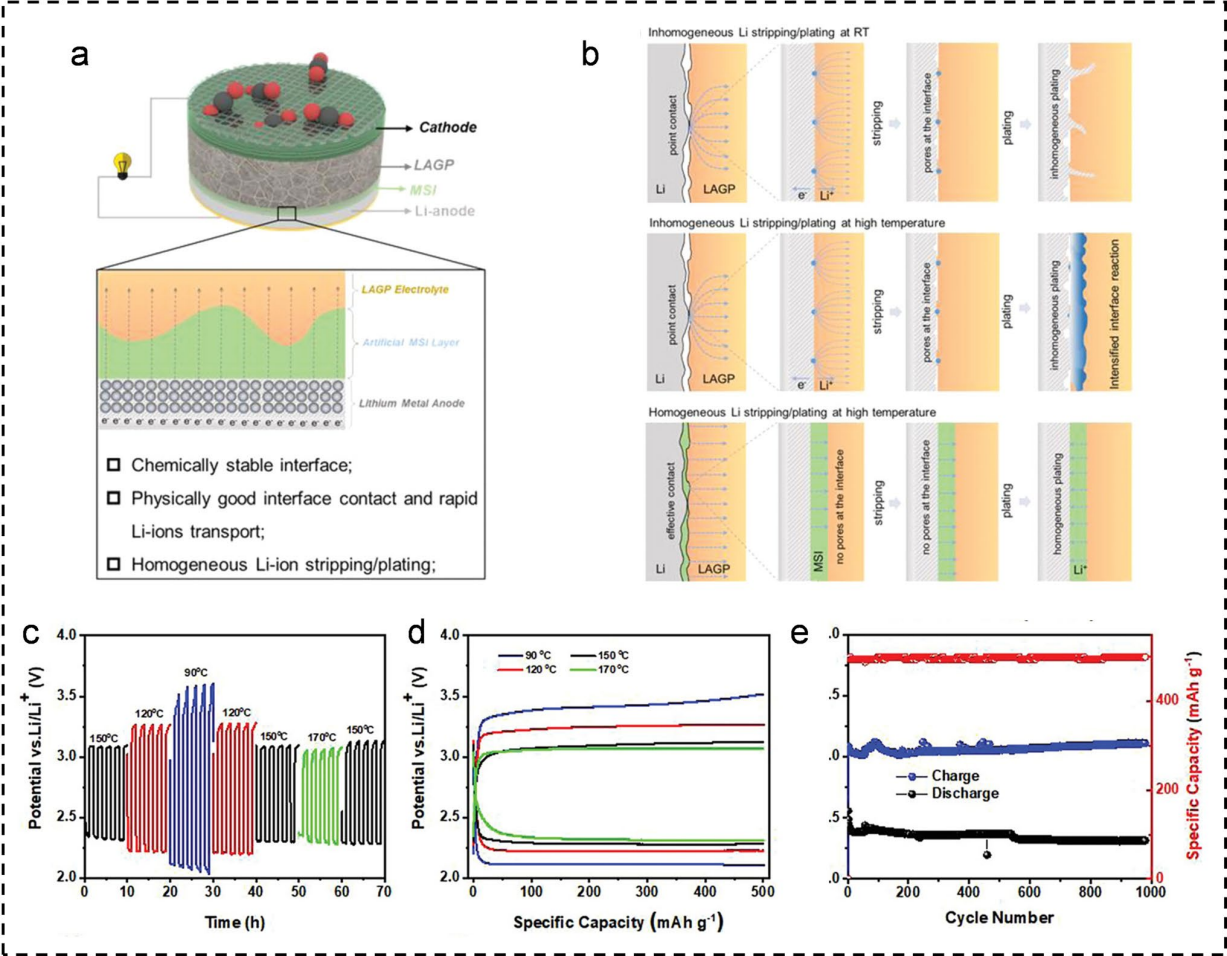
**a** Fabrication of solid-state Li–CO_2_ batteries with MSI layer. **b** Morphological changes of initial LAGP and LAGP with MSI layer during lithium stripping/plating. **c**-**d** Electrochemical performance operating from 90 to 170 °C with limited capacity of 500 mAh g^−1^ at 500 mA g^−1^. **e** Cycle performance of the Li-CO_2_ battery with MSI layer at 150 °C

In addition, molten electrolytes show significant advantages in M-CO_2_ batteries that can operate at HT, especially in terms of improving interface stability and enhancing battery safety [76]. First, the molten electrolyte is able to maintain excellent ionic conductivity at HT, thus ensuring stable battery performance over a wider temperature range. Secondly, the molten electrolyte can form a solid physical contact between the metal anode and the solid electrolyte, which reduces the interface impedance, effectively prevents the interface reaction, and inhibits the thermal runaway phenomenon. This not only extends the cycle life of the battery, but also significantly improves its safety. Kyungeun Baek et al. developed a Li-CO_2_ battery utilizing a nitrate-based molten salt electrolyte and employing nano-Ru as the cathode catalyst [76]. The incorporation of the molten salt electrolyte enhanced the CO_2_ capture rate, effectively mitigating overpotential during charging and discharging processes. With the synergistic effect of the nano-Ru catalyst, the battery exhibited stable cycling for 300 cycles even under HT (150 °C) and high current density (10.0 Ag^−1^), achieving a peak power density of 33.4 mW cm^−2^. Subsequently, Xu’s team effectively alleviated the problem of rapid degradation at the lithium anode and solid-state electrolyte interface under HT by constructing a stable and highly ion-conductive molten salt interface (MSI), building upon their previous research. The lithium metal batteries successfully operated within a high-temperature range of 90 to 170 °C—temperatures surpassing the decomposition point of organic electrolytes (90 °C) yet remaining below the melting point of lithium metal (~ 180 °C) [29]. The incorporation of the MSI layer in the battery structure, as depicted in Fig. 8a, not only enhances the physical contact and chemical stability between the lithium metal and solid-state electrolyte but also effectively mitigates safety concerns such as fire hazards at elevated temperatures. Even at elevated temperatures, the MSI layer effectively enhanced interface contact, suppressed interface reactions, and mitigated thermal runaway between the lithium anode and Li_1.5_Al_0.5_Ge_1.5_P_3_O_12_ (LAGP) electrolyte. As a result, high-temperature symmetric cells exhibited ultra-low interface impedance (~ 15 Ω) and low overpotential (~ 15 mV) during discharge/charge cycles. Additionally, the LAGP electrolyte with the MSI coating exhibited an ultra-flat and continuous surface, enabling uniform lithium stripping/plating during cycling (Fig. 8b). The Li symmetric cell demonstrated ultra-long cycling stability for 600 h at 150 °C and 0.1 mA cm^−2^. Meanwhile, the solid-state Li–CO_2_ battery assembled with a metallic Ru catalyst showed good stability across the temperature range of 90 to 150 °C, exhibiting excellent cycling stability even at 150 °C (980 cycles at 500 mAh g^−1^) (Fig. 8c-e). This solid-state Li–CO_2_ battery can also operate at RT with high efficiency by directly capturing solar energy, which undoubtedly is an efficient and economical carbon reduction technology.

##  Summary and Outlook

### Opportunities

The wide-temperature M-CO_2_ battery holds significant research value across multiple domains.

1. Environmental protection and carbon reduction: The wide-temperature M-CO_2_ battery demonstrates the potential to convert CO_2_ into valuable products or directly generate electricity, thereby contributing to greenhouse gas emission reduction and mitigating global climate change. Its development offers novel solutions for carbon capture and utilization.
2. Efficient storage and utilization of renewable energy: By integrating with renewable energy systems, the wide-temperature M-CO_2_ battery provides efficient energy storage solutions, crucial for balancing intermittent supply from renewable sources. This enhances overall energy utilization efficiency and promotes the advancement of clean energy technologies.
3. Expanding application scope: With its capability to operate in a broad temperature range, the wide-temperature M-CO_2_ battery is well-suited for extreme environments such as space exploration, polar research, and military applications. It delivers reliable energy solutions tailored to these fields.

### Challenges and Strategies

M-CO_2_ batteries exhibit similarities to the M–O_2_ batteries more extensively studied; however, they encounter more intricate challenges, particularly under extreme temperature conditions [19, 77–79]. Specifically: (1) Gas transport and reactivity: The dissolution and transport capacity of CO_2_ in the electrolyte is significantly lower than that of O_2_. Even at complex three-phase interfaces, O_2_ transmission is smoother and less affected by temperature changes. At the same time, the high chemical inertness of CO_2_ (e.g., C = O bond energy up to 750 kJ mol^−1^) poses challenges for its reduction compared to O_2_. At LT, although the solubility of CO_2_ improves, limited diffusion slows down reaction kinetics. Conversely, at HT, while reactivity increases, gas diffusion capacity decreases thereby limiting overall reaction efficiency. (2) The complexity of the reaction path and by-products: CRR involves complex multielectron transfer processes and the formation of multiple intermediates, such as oxalates and formates. In contrast, the oxygen reduction/evolution reaction (ORR/OER) follows a more direct and simplified path. At lower temperatures, solid-state by-products like Li_2_CO_3_ tend to accumulate on the electrode surface, leading to passivation and increased impedance. On the other hand, at higher temperatures, active intermediates may exacerbate side reactions, causing damage to the electrode material and impacting battery stability. (3) Product reversibility and battery polarization: In the CER process, elevated temperatures facilitate the oxidation and desorption of solid or liquid carbon-based compounds (e.g., Li_2_CO_3_), thereby reducing their deposition on the electrode surface. However, at LT, these products exhibit limited desorption capability, leading to increased polarization and potential failure. For example, in M-CO_2_ batteries, Li_2_CO_3_ demonstrates enhanced stability at LT but becomes challenging to decompose during charging, resulting in the accumulation of irreversible reactions and a decline in cycling performance. Conversely, Li_2_O_2_ formed in M–O_2_ batteries exhibits superior reversibility as it readily undergoes reduction to O_2_ during charging.

Overall, the challenges associated with M-CO_2_ batteries primarily revolve around the inertia of CO_2_, the complexity of the reaction pathways, and the irreversibility of the products. At LT, M-CO_2_ batteries are particularly vulnerable to limitations imposed by reaction kinetics and the accumulation of by-products. Conversely, at elevated temperatures, while reaction rates may increase, there is a heightened risk that side reactions and intermediates will adversely affect battery performance and stability. Next, specific optimization strategies will be summarized from three aspects: enhancing electrolyte stability and electrolyte–electrode interface integrity; regulating metal nucleation and deposition behaviors; and optimizing CRR/CER processes.

#### For Electrolytes and Interfaces

1. Low-temperature optimization: At LT, the primary challenges would be reduced ionic conductivity, increased viscosity, and potential electrolyte freezing. First, it is crucial to select a single or mixed solvent with a low freezing point, such as an ether solvent, to ensure effective ion migration. Additionally, electrolyte additives or high concentrations of salts can contribute to the formation of a stable SEI film, which can reduce the migration energy barrier of M^+^ and facilitate ion transport.
2. High-temperature optimization: At HT, the main concerns would be liquid electrolyte decomposition, increased side reactions at the interface, and potential electrolyte volatility. Therefore, the selection of thermally stable salts and high boiling point solvents is a primary consideration, with solid electrolytes also warranting attention. Furthermore, additives capable of forming stable SEI and cathode electrolyte interface (CEI) films should be considered to effectively protect the interface by creating a high-density polymer film on the surface, thereby mitigating the occurrence of side reactions.

#### For Metal Deposition/Stripping

1. Low-temperature optimization: One of the primary challenges encountered in the deposition and precipitation of metals under LT conditions is the significant restriction of the deposition kinetics, which readily leads to uneven deposition and the formation of dendrites. To this end, by adjusting the solvation structure of the M^+^ in the electrolyte, preforming or in situ constructing a SEI layer, and adopting three-dimensional (3D) anodes, the uniform nucleation and growth of the metal can be effectively facilitated, thereby reducing the generation of dendrites. Among them, the 3D anode is capable of buffering stress changes during the deposition process, enhancing the cycling stability of the battery. Meanwhile, the SEI layer can offer outstanding interface stability over a wide-temperature range, contributing to the reduction of interface impedance and the enhancement of the controllability of the metal deposition process.
2. High-temperature optimization: Owing to the acceleration of reaction kinetics, metals are susceptible to uncontrolled deposition in high-temperature environments, which likewise gives rise to the rapid growth of dendrites, thereby triggering short circuits or battery failure. Similar to the strategies employed under low-temperature conditions, the deposition of metals at HT can be effectively regulated by adjusting the solvation structure and constructing the SEI layer. Furthermore, it is feasible to add a protective coating with high-temperature stability on the surface of the anode to enhance the stability of the interface, such as ceramic or polymer materials.

#### For CRR/CER

1. Low-temperature optimization: At LT, the kinetics of both CRR and CER are significantly decelerated. Particularly for organic-based M-CO_2_ batteries, the reduction reaction typically yields solid-phase products, and their decomposition becomes more challenging at LT. In such circumstances, on the one hand, it is essential to guarantee that the electrolyte possesses a relatively high CO_2_ solubility and transport capacity. On the other hand, the selection of highly efficient dual-function catalysts with excellent temperature adaptability, such as nanostructured noble metal catalysts (e.g., Ru, Ir, etc.) and their composite catalysts, is of paramount importance. Additionally, the electrode surface area can be augmented through structural design to enhance the dispersion of the catalyst active sites, thereby elevating the contact efficiency with CO_2_ and simultaneously ensuring the uniform deposition of solid-phase reduction products like Li_2_CO_3_. Moreover, external field regulation approaches, such as light energy assistance and pressure modulation, can be contemplated to facilitate CRR/CER processes at LT.
2. High-temperature optimization: At HT, although the reaction dynamics are improved, a series of problems also arise. For example, degradation of catalysts at HT and changes in the active site may lead to capacity decline; at the same time, side reactions involving CO_2_ and its intermediate state will also intensify. Therefore, the selection of catalysts with HT stability has become a priority. In addition, by introducing a high-temperature resistant coating on the electrode surface, its structural integrity and reaction stability at HT can be enhanced. In order to avoid the reaction failure caused by local accumulation of the solid-phase product, the design of the positive electrode with porous structure is conducive to the uniform nucleation and growth of the product, and can also improve the heat dissipation performance of the electrode to avoid the catalyst failure caused by local overheating. In addition, the direct introduction of a thermal management system to control the internal temperature of the battery is also an effective means to ensure the stable operation of the battery in a HT environment.

In summary, to achieve optimized performance for M-CO_2_ batteries across a wide-temperature range (such as from sub-zero to over 100 °C), a combination of strategies is essential. Liquid electrolytes perform well at medium-to-low temperatures, facilitating M^+^ and CO_2_ transport and enhancing CO_2_ reduction reactions. However, at extremely LT, liquid electrolytes may freeze or lose conductivity, increasing interface impedance and leading to battery failure. In contrast, while solid electrolytes provide good thermal stability and ion conductivity at HT, they face challenges like high interface impedance at LT, limiting their performance. Therefore, relying on a single type of electrolyte is insufficient across wide-temperature ranges. A mixed solid–liquid electrolyte system or the use of hybrid SEI layers that dynamically adjust to varying conditions can ensure efficient ion transport and structural integrity. Additionally, the incorporation of temperature-adaptive materials, such as flexible coatings and additives, further improves the adaptability of the battery to temperature changes. Porous electrode designs, along with high-temperature resistant coatings, help maintain stable reactions and control solid-phase product growth. Furthermore, external field control mechanisms and a comprehensive thermal management system are critical for maintaining the battery’s optimal internal temperature.

### Directions

**Fig. 9:**
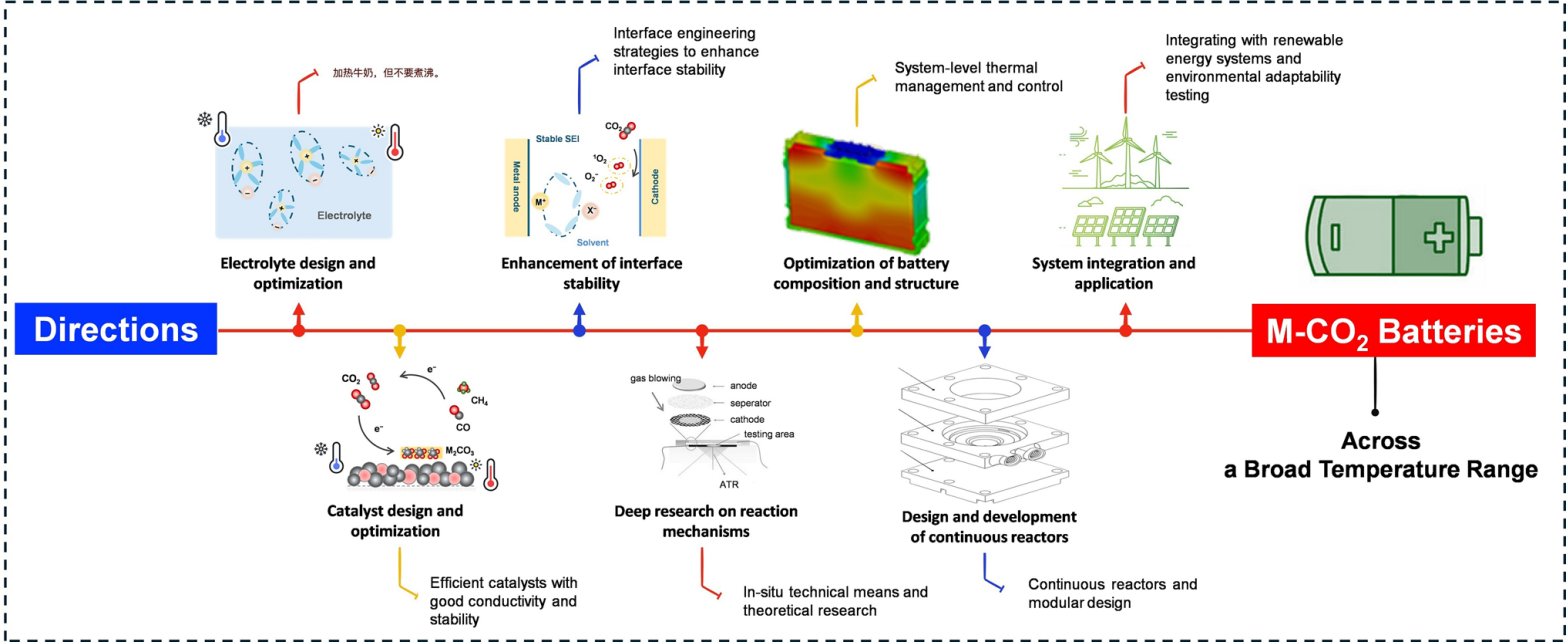
“Roadmap” of a guideline for future directions of wide-range temperature M-CO_2_ batteries

Future research on M-CO_2_ batteries in a wide-temperature range needs to focus on the following aspects (Fig. 9):

- Electrolyte design and optimization: To address the issue of reduced electrolyte conductivity at LT, researchers must develop stable electrolytes with high ion conductivity across a broad temperature range. By further optimizing the composition and structure of the electrolyte, improvements can be made to its conductivity and freeze resistance, ensuring stability over a wide-temperature range and enabling efficient battery operation under various temperature conditions.
- Catalyst design and optimization: In a wide-temperature range, highly efficient and stable catalysts are crucial for battery performance. It is necessary to explore the use of materials with good conductivity and stability as the cathode catalyst, and to improve the material structure for different temperature conditions to enhance the electrode's reaction activity and cycling stability.
- Enhancement of interface stability: To address the problem of weak dehydration at the electrode/electrolyte interface in LT conditions, interface engineering strategies are adopted to enhance interface stability and catalytic activity, thereby improving the energy conversion efficiency and cycle life of the battery.
- Deep research on reaction mechanisms under variable temperatures: Considering the complexity of the CO_2_ battery reaction mechanisms, a deep understanding of the reaction mechanisms at different temperatures and optimization of reaction conditions are key to improving battery efficiency, including the cathode CRR/CER, the deposition/evolution of metals at the anode, and the solvation effect in the electrolyte. The future requires in situ technical means and theoretical research to deeply understand the reaction mechanisms involved in M-CO_2_ batteries at different temperatures, thereby guiding the optimization of reaction conditions and improving the selectivity and efficiency of the reaction.
- Optimization of battery composition and structure: By optimizing the battery structure through the rational coupling and decoupling of mechanical and electrical properties, it is possible to enhance the overall performance of the battery. For instance, stress engineering strategies can be employed to regulate the surface electronic structure and reaction activity of the catalyst, while designing electrode structures with high specific surface areas and excellent conductivity can improve reaction efficiency. Besides, to address the problem of battery performance being affected by extremely LT, system-level thermal management and control research is conducted to improve the overall performance and reliability of the battery in LT environments.
- Design and development of continuous reactors: The design of continuous reactors aims to enhance the scale and efficiency of CO_2_ reduction reactions. Flow battery systems can be developed to achieve continuous recirculation of CO_2_ and electrolyte, thereby improving reaction efficiency. Additionally, modular design can be adopted to enable flexible assembly and expansion of the battery system in order to meet diverse application requirements.
- System integration and application: Integrating wide-temperature M-CO_2_ batteries with renewable energy systems, such as combining with solar photovoltaic systems, the wide-temperature characteristics can enable the battery to operate normally in both high-temperature sunlight and low-temperature nights. In addition, it is highly necessary to conduct environmental adaptability tests on the battery in an experimental environment to simulate extreme conditions (including extremely cold and hot temperatures) to promote the practical application of wide-temperature batteries.

In summary, propelled by the low-carbon economy, M–CO_2_ batteries exhibit significant potential for commercialization. With advancements in materials science and electrocatalytic technology, the prospects of M–CO_2_ batteries in mobile energy applications, such as electric vehicles and portable devices, are also promising. However, the large-scale commercialization of M–CO_2_ batteries necessitates overcoming a range of technical and economic challenges. Firstly, it is essential to reduce material costs by developing cost-effective and efficient catalysts to satisfy the increasing market demand for affordable energy storage solutions. Secondly, the stability and energy efficiency of the reaction require further enhancement, with a focus on ensuring long-term durability while maintaining efficient CO_2_ conversion. The durability of the materials and the low-temperature performance of the electrolytes are also critical factors. Through optimization of catalysts, electrolytes, and battery architecture, it is possible to significantly improve battery performance across a range of temperature conditions. Thirdly, comprehensive assessments for environmental adaptability represent a crucial domain of research. M–CO_2_ batteries must be rigorously evaluated in both laboratories and real-world applications, especially under extreme conditions (like LT or HT) to ensure stable operation across various environments. Finally, the commercialization trajectory of M–CO_2_ batteries must be intricately aligned with both policy frameworks and market demand. The carbon–neutral policies implemented by various nations have significantly accelerated the adoption of this innovative class of batteries. The M–CO_2_ battery is poised to play a pivotal role in the future of energy storage, provided it aligns with policy directives, satisfies market demands, and enhances its performance through technological advancements.
